# Generation of murine tumour-reactive T cells by co-culturing murine pancreatic cancer organoids and peripheral blood lymphocytes

**DOI:** 10.1016/j.bbrep.2022.101365

**Published:** 2022-10-09

**Authors:** Alberto D'Angelo, Kensuke Shibata, Masayuki Tokunaga, Makoto Furutani-Seiki, Stefan Bagby

**Affiliations:** a Department of Biology and Biochemistry, University of Bath, Bath, BA2 7AY, United Kingdom; b Department of Biology and Biochemistry, University of Yamaguchi, Ube, Japan

**Keywords:** Pancreatic cancer, Tumour microenvironment, Co-culture, Cancer organoids, Murine model, Peripheral blood mononuclear cell

## Abstract

Pancreatic ductal adenocarcinoma (PDAC) is commonly diagnosed at a late stage and becomes resistant to several treatments. Significant clinical effects have been reported for cancer immunotherapies on a subset of patients diagnosed with epithelial cancers. Cancer organoid co-culture with autologous peripheral blood lymphocytes offers an innovative immunotherapeutic approach that is increasingly being tested, although there is a lack of cutting-edge platforms enabling the investigation of cancer-T cell interactions for individual patients. In this study, a pancreatic cancer organoid culture from a genetically engineered pancreatic cancer murine model was established and co-cultured with autologous peripheral blood lymphocytes to induce a tumour-specific T cell response to pancreatic cancer. Co-culturing autologous peripheral blood lymphocytes with cancer organoids can be an effective strategy to enrich tumour-reactive T cells from the peripheral blood of murine models; this approach could potentially be transferred to humans. Co-culture of peripheral blood lymphocytes and cancer organoids could provide an unbiased approach to evaluating the sensitivity of tumour cells to T cell-mediated priming on an individual patient level.

## Highlights

• Pancreatic cancer organoids co-cultured with PBMCs can establish an individualized ex-vivo model system to support T cell-based therapies.
• Granzyme B intracellular staining showed increased tumour reactivity in T cells co-cultured with cancer organoids compared to healthy controls.
• CD137 staining showed that the T cell responses induced by co-culture were specific to the cancer organoids.

## 1 Introduction

Pancreatic ductal carcinoma (PDAC) is an aggressive malignancy with a poor overall survival rate of approximately 9% at five-year follow-up [15]. Only 10–20% of patients are eligible for curative surgical resection. 80–90% of patients are diagnosed at a local advanced or metastatic stage, making them eligible for either adjuvant or neoadjuvant cytotoxic chemotherapy followed by surgical resection [18]. Immunotherapy, namely a type of therapy that uses substances to stimulate or suppress the immune system, is expected to significantly change the treatment of cancer patients. Immunotherapeutic approaches include monoclonal antibodies, antitumour vaccines, immune system modulators and immune checkpoint inhibitors, and adoptive transfer therapy. Adoptive transfer therapy has shown significant responses in patients, mainly among those with melanoma and early-stage cervical cancer [12,16]. Despite these promising outcomes, a large number of patients fail to respond to existing immunotherapies. Treatment failure occurs due to a range of reasons, including the expression of alternative immune checkpoints and poor antigen presentation [14].

The numerous strategies that cancers use to elude the immune system make it challenging to predict whether a patient will benefit from immunotherapy agents, elucidate any underlying resistance mechanism and determine the best immunotherapy approach for overcoming any resistance. T cell repertoire and cancer mutation profile are unique to every patient. Consequently, a more effective therapeutic strategy to exploit the patient's anti-tumour immunity requires a personalized experimental evaluation of immune responses that should be applicable within the clinical timescale [13].

Ex vivo strategies to assess T cell-tumour interaction have mainly focused on melanoma due to the number of available patients, the ease of tumour biopsy and the availability of techniques for the expansion of tumour infiltrating lymphocytes (TILs) [12]. With the current clinical use of immunotherapies for epithelial cancers [2], however, it is important to develop technologies to analyse T cell-mediated tumour effects on different tumour types. So far, several factors have hampered progress towards this goal, including difficulty in extracting tumour-reactive T cell populations and limited success rates in establishing epithelial cancer cell lines such as for colorectal and lung cancer [3,20].

In this study, pancreatic cancer organoids were established from mouse models and were co-cultured with peripheral blood mononuclear cells (PBMCs). Tumour organoids are three-dimensional tumour cell cultures retaining the mutational burden and histological features of the primary tumour. Organoids can be obtained from needle biopsies or surgical tumour resection. The aim was to establish whether tumour-reactive T cells can be established by co-culturing PBMCs with tumour organoids and also whether T cell responses induced by organoid co-culture are tumour-specific or should be considered artefacts. Differentiation of PBMCs into T cells provides an easily accessible alternative to peripheral isolated lymphocytes. This approach could be helpful towards achieving patient-specific medicine; in theory, tumour-derived organoids could be used to establish personalized ex vivo model systems to support T cell-based therapies. These organoids could also shed light on the interactions between malignant cells and T cells.

A proof of concept is provided that co-culturing tumour organoids with PBMCs is a sound and unbiased approach to obtaining tumour-reactive T cells that could potentially suppress tumour cells. More broadly, the findings corroborate the idea that this strategy can affect the priming of T cells against tumour cells towards personalized immunotherapy.

## 2 Methods

### 2.1 Experimental murine model

Two pairs (2 males and 2 females) of C57BL/6 genetically engineered mouse models (GEMMs) of pancreatic cancer (LSL-Kras^G12D^ Trp53^lox/+^ Pdx1-Cre (KPC)) were brought from Nihon University School of Medicine of Tokyo (Japan) and were harvested at Yamaguchi University's mouse facility. All animal studies were conducted in compliance with the international guidelines for the care and use of laboratory animals and were approved by the Yamaguchi University Institutional Review Board.

### 2.2 Tumour collection

One male GEMM mouse was sacrificed by cranial dislocation after six months; the sample collection time point was collectively agreed once the mouse developed a clear and touchable mass in the abdomen and was no longer amenable to movement. The pancreatic tumoral mass was extracted and immediately minced into 10 mm^3^ fragments with surgical scissors in a solution containing Advanced Dulbecco's Modified Eagle's Medium (adDMEM)/F12 medium (GIBCO) plus 100 mg/mL collagenase II (Sigma-Aldrich), 10 μM Y-27632 (Sigma-Aldrich) and 0.06 mg/mL DNase I (Sigma-Aldrich).

### 2.3 Establishment of tumour organoids from resected material

Following 30 min incubation at 37°C, the cancer cell suspension was transferred into a 50 mL tube containing 10 mL of adDMEM/F-12 medium (Gibco), pipetted up and down strongly and subsequently centrifuged at 200 g for 5 min at 4°C. Ultimately, cells were counted using a hemocytometer, resuspended in Matrigel Growth Factor Reduced (BD) (3 × 10^4^ cells per 10 uL medium) and 20 uL per well of Matrigel resuspension was added in a 48-well culture plate. The plate was placed into a 37°C incubator for 30 min to allow the Matrigel to solidify and 250 mL of organoid medium was then added and the nascent organoids were incubated at 37°C with 5% CO_2_. Pancreatic organoid medium consisted of adDMEM/F-12 medium (Gibco) supplemented with 50% Wnt3a medium (Protocol 1) [5], 10% R-spondin medium (Protocol 2) [5], 10 ng/mL Noggin medium (Protocol 3) [1], 1/500 Primocin (Invivogen), 10 mM HEPES (Gibco), 2 nM Ultraglutamine (Lonza), 500 nM A83-01 (Tocris), 1 nM *N*-acetyl-l-cysteine (Sigma-Aldrich), 2% B27 supplement (Gibco), 50 ng/mL FGF-2 (Peprotech), 50 ng/mL EGF (Peprotech) supplemented with 10 μM Y-27632 (Sigma-Aldrich).

### 2.4 Passaging organoids

When confluent or the diameter exceeded 300 μm, organoids were passaged. For this passaging step, the organoid medium was aspirated and organoid-Matrigel drops were resuspended in pre-warmed TrypLE Express (Gibco) (1 mL per well), followed by a 5–15 min incubation at 37°C, resuspending with a P1000 pipette every 5 min. Organoid dissociation into single cells was confirmed under a microscope. Afterwards, adDMEM/F-12 (Gibco) medium was added five-to ten-fold to inhibit TrypLE. The cell suspension was then centrifuged at 300 g for 5 min at 4°C, the supernatant was removed and cells were resuspended in Matrigel as described in the previous paragraph.

### 2.5 Isolation of murine PBMCs from blood

The cardiac puncture technique was used to obtain approximately 300 μL of blood from a euthanised and sacrificed GEMM mouse and from a healthy mouse with the same genetic background (C57BL/6). Blood was collected in a 15 mL tube containing 100 μL heparin and a volume equal to the blood volume (in this case, 300 μL) of 0.9% NaCl and then layered onto 3 mL Lymphoprep solution (StemCell) and centrifuged for 20 min at 20°C. The formed PBMC layer was subsequently collected in a separate tube and washed twice with 10 mL of 2% RPMI 1640 (Gibco). PBMCs were counted using an automated cell counter.

### 2.6 Organoid isolation for co-culture of tumour cells and PBMCs

When organoids reached approximately 150 μm diameter and were suitable for co-culture, medium from a single 48-well plate was aspirated and 1 mL per well of pre-heated Dispase (Sigma-Aldrich) (2 mg/mL in PBS) was added to enable isolation of organoids from Matrigel (BD). After being resuspended by gentle pipetting, organoids were incubated for 15 min at 37°C. The organoid suspension was transferred into a 15 mL falcon tube with 0.5 M EDTA (100 μL for every 1 mL dispase) and filled up to 10 mL with PBS. Organoids were then centrifuged at room temperature for 5 min at 300 g, resuspended in organoid culture medium and plated (1 mL per well) in a 48-well plate. To prevent cell death, 10 μM Y-27632 (Sigma-Aldrich) was added to the organoid before culturing in a 48-well plate for 24 h at 37°C.

### 2.7 Co-culture preparation

24 h before the co-culture experiment, tumour organoids were stimulated overnight with 200 ng/mL INFγ to enhance antigen presentation. Meanwhile, a 96-well U-bottom plate was coated with 5 μg/mL anti-CD28 (50 μL per well) to provide co-stimulatory signals during co-culture. The 96-well U-bottom plate was subsequently wrapped in Parafilm and incubated for 24 h at 4°C. Furthermore, 2-3 x 10^6^ PBMCs were resuspended in T cell culture medium [adDMEM/F-12 (Gibco) supplemented with 2 nM UltraGutamine (Lonza), 1:100 penicillin/streptomycin, 10% human serum AB (Sigma-Aldrich)] supplemented with 150 U/mL IL-2 (Novartis) and incubated overnight at 37°C with a slightly opened lid wrapped in Parafilm to enable gas exchange.

### 2.8 Co-culture experiment

Organoids incubated with INFγ were centrifuged (300 g, 5 min at RT), resuspended in 1 mL TrypLE (Gibco) and incubated for 15 min at 37°C. Dissociated organoids were centrifuged and washed in PBS and then resuspended in T cell culture medium. The resulting single-cell organoid suspension was counted using a hemocytometer and resuspended at 5 × 10^4^ cells per mL in T cell culture medium. Meanwhile, PBMCs were counted using an automated cell counter, washed in PBS and resuspended at 1 × 10^6^ cells per mL in T cell culture medium supplemented with 300 U/mL IL-2 (Novartis) (2x concentrated). Equal volumes (100 μL) of PBMCs and dissociated organoids were mixed for a PBMC:tumour cell ratio of 20:1.200 μL per well of PBMC-dissociated organoid suspension mix was then plated and incubated at 37°C for 10 days. The co-culture medium was refreshed every 2–3 days.

### 2.9 Healthy pancreas organoids

Along with the expansion of tumour organoids, a healthy pancreatic organoid cell culture was established. A mouse with the same genetic background (C57BL/6) was sacrificed, the pancreatic tissue was removed and healthy pancreatic organoids were expanded using the same protocol as used for pancreatic cancer organoids.

### 2.10 Flow cytometry for downstream assays

Following co-culture, cells were immediately evaluated for tumour reactivity using Granzyme B and CD137 expression as read-out. As a positive control, an equal volume and number of PBMCs were incubated with phorbol 12-myristate 13-acetate (PMA)/Ionomycin (Sigma-Aldrich) solution at 37° for 4 h. As a negative control, an equal volume and number of PBMCs were incubated with healthy organoids at 37° for 10 days and 24 h for Granzyme B and CD137 assays, respectively.

#### 2.10.1 Tumour reactivity assay using Granzyme B as read-out

After 10 days of incubation, each co-culture well was resuspended at 2 × 10^6^ cells per mL in 400 μL T cell medium and split into two aliquots (200 μL each). Cells were washed twice in FACS buffer and stained with the following antibodies (extracellular staining): FITC-CD8a (1:80)(Peprotech), Per55-I-A/I-E (1:200) (Peprotech), APC-TCRb (1:40) (Peprotech), at 4°C for 20 min. Cells were then washed twice in FACS buffer, fixed and stained (intracellular staining) for Granzyme B (PE-conjugated anti-GrB) (Peprotech), using the Cytofix/Cytoperm kit (BD), according to the manufacturer's instructions. PBMCs co-cultured with healthy organoids served as a negative control whereas PBMCs stimulated with 50 ng/mL PMA (Sigma-Aldrich) and 1 μg/mL Ionomycin (Sigma-Aldrich) served as a positive control; half of the contents of each co-culture well was incubated with PMA/Ionomycin solution for 5 h on ice.

#### 2.10.2 Tumour reactivity assay using CD137 expression as read-out

In a parallel co-culture experiment, after 24 h incubation, co-cultured T cells were resuspended at 1 × 10^6^ cells per mL, washed twice in FACS buffer and stained with the following antibodies: PE-anti-CD137 (1:30) (Peprotech), FITC-CD8a (1:80) (Peprotech), Per55-I-A/I-E (1:200) (Peprotech), APC-TCRb (1:40) (Peprotech). PBMCs co-cultured with healthy organoids served as a negative control whereas PBMCs stimulated with 50 ng/mL PMA (Sigma-Aldrich) and 1 μg/mL Ionomycin (Sigma-Aldrich) served as a positive control.

## 3 Results

### 3.1 Tumour development

**Fig. 1:**
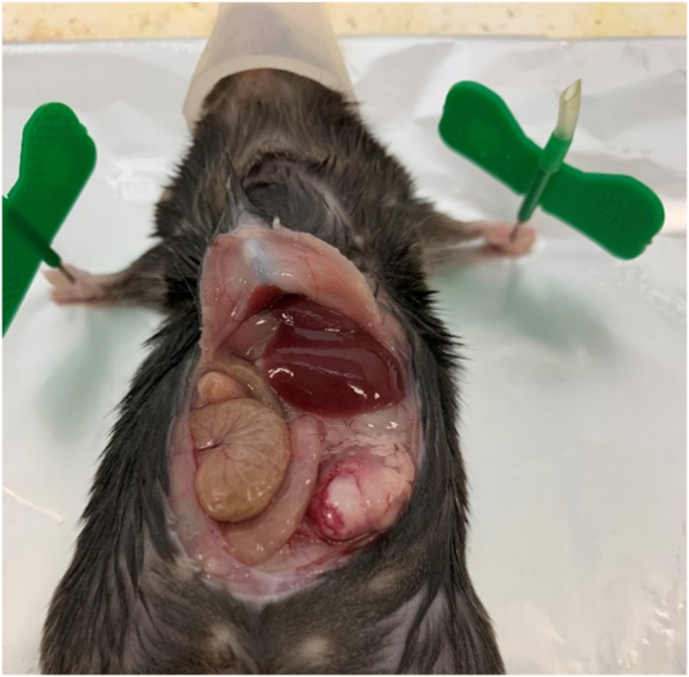
Sacrificed GEMM with pancreatic cancer in the left flank (red arrow). (For interpretation of the references to color in this figure legend, the reader is referred to the Web version of this article.)

**Fig. 2:**
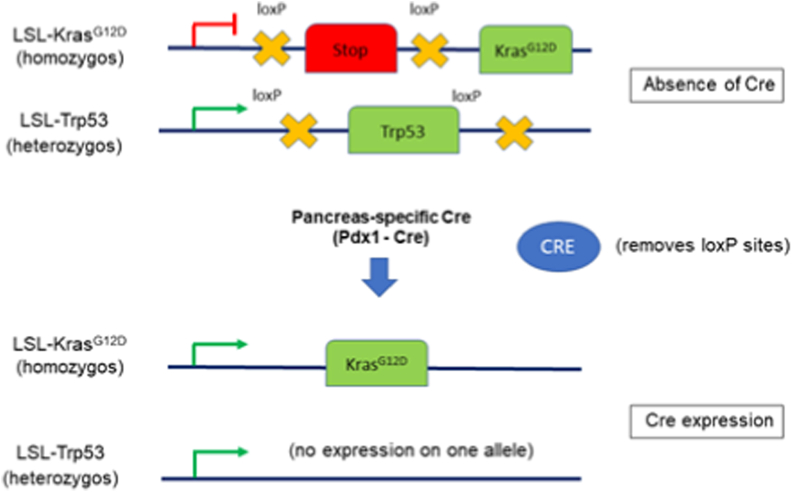
The genetic background of the pancreatic cancer GEMM used here. The conditional expression of mutant Kras^G12D^ and Trp53 is controlled by a pancreas-specific Cre (Pdx1-Cre in the model described here). In the absence of Cre, a transcriptional and translational STOP cassette flanked by loxP sites (LSL) silences the expression of mutant Kras^G12D^ whereas Trp53 is expressed as normal. When Cre is expressed in the pancreas, loxP sites are excised. The homozygotic expression of mutant Kras and the heterozygotic expression of Trp53 in the murine pancreas results in pre-invasive pancreatic intraepithelial neoplasia (PanIN), which progress to pancreatic ductal adenocarcinoma (PDAC).

LSL-Kras^G12D^ Trp53^lox/+^ Pdx1-Cre mice developed a well differentiated pancreatic cancer (Fig. 1). In these GEMM mice, the conditional expression of mutant Kras^G12D^ and heterozygotic knockout of Trp53 is controlled by a pancreas-specific Cre recombinase which, in turn, is under the control of a Pdx1 promoter. In the absence of Cre, a transcriptional and translational STOP cassette flanked by loxP sites (LSL) silences the expression of mutant Kras^G12D^ whereas expression of Trp53 is maintained on both alleles. When Cre is expressed in the pancreas, the STOP cassette is excised and the mutant Kras^G12D^ alleles are expressed and Trp53 is knocked out on a single allele (Fig. 2). This results in pre-invasive pancreatic intraepithelial neoplasia (PanIN) at approximately six weeks of age; this progresses to pancreatic ductal adenocarcinoma (PDAC), with a latency ranging from four to eight months.

### 3.2 Pancreatic organoid line authentication

**Fig. 3:**
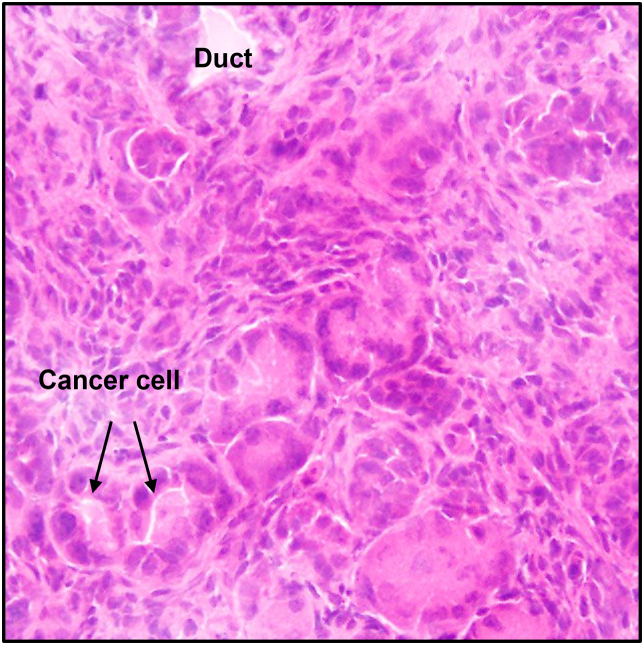
Histological analysis of GEMM pancreatic tissue. H&E staining revealed glandular ductal cancer cells (black arrows).

**Fig. 4:**
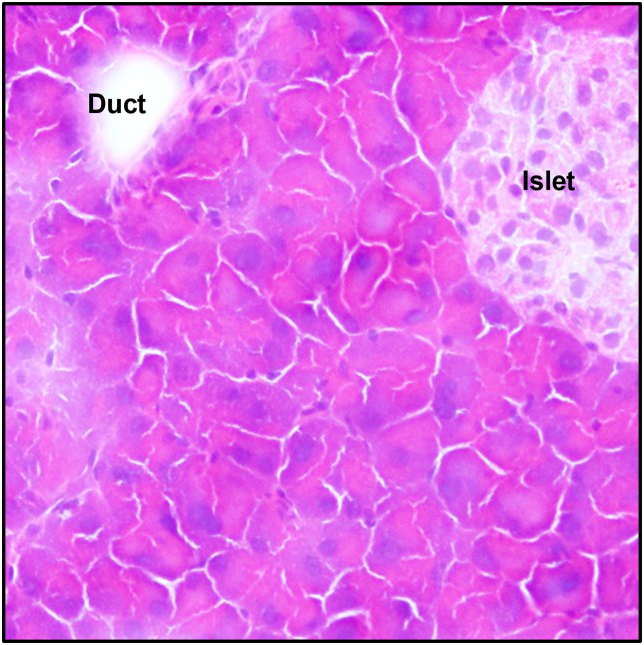
Histological analysis of healthy pancreatic tissue from healthy mouse with the same genetic background (C57BL/6). H&E staining revealed normal glandular cells.

Using histological analysis, we established a discriminating selection of cancerous cell-derived organoids. After hematoxylin and eosin (H&E) staining, histological analysis revealed that the tumours in this study were carcinomas, predominantly ductal adenocarcinomas (PDAC), defined by the presence of neoplastic glandular (ductal) cells in a dense fibrous stroma (Fig. 3). As a comparison, we also stained a healthy area of the pancreas (Fig. 4) with an islet of Langerhans in the top right corner.

### 3.3 Establishment of pancreatic cancer organoids and healthy pancreas organoids

**Fig. 5:**
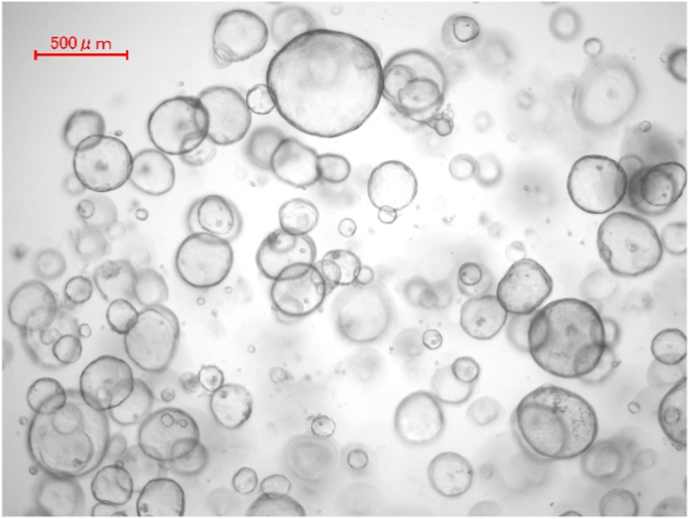
Pancreatic cancer organoids after 36 h-culturing in organoid medium and several passaging steps.

**Fig. 6:**
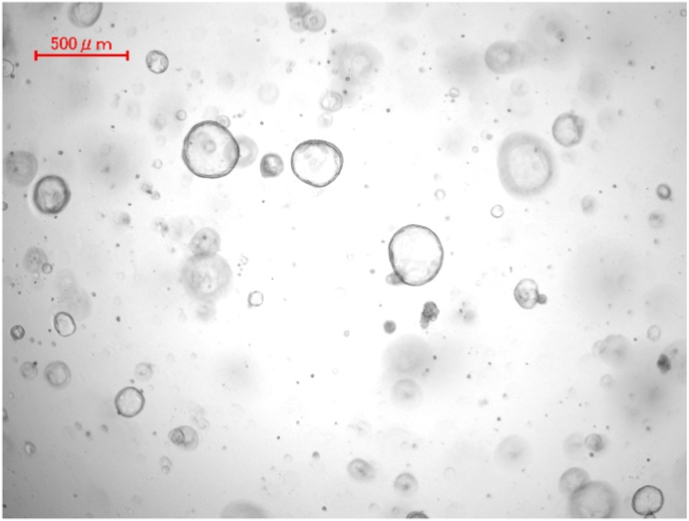
Pancreatic organoids from healthy mouse after 36-h culturing in organoid medium and several passaging steps.

Cancerous cells from pancreatic cancer mouse models, and cells from normal pancreatic tissue, embedded in Matrigel and organoid medium, started developing organoids - defined as a three-dimensional (3D) cellular cluster derived exclusively from primary tissue - after 3–5 days of incubation (Fig. 5, Fig. 6). The success rate of organoids from these sources was approximately 40%. Organoids were expanded every 2–4 days, passaging at 1:2 to 1:5 split ratios. Organoids can be cryopreserved at −80°C in biobanks and recovered from frozen stocks and expanded for several months without loss of proliferative capacity. In the initial experiment, the outgrowth of normal pancreas organoids and those derived from cancerous cells was as expected.

### 3.4 Induction of tumour reactivity in circulating T cells by co-culture with autologous tumour organoids

**Fig. 7:**
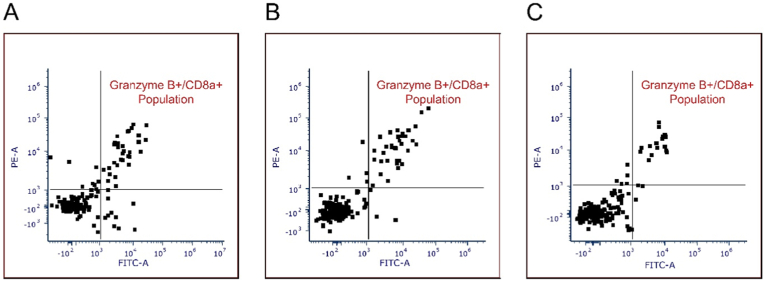
FACS analysis of tumour reactivity of PBMCs-turned-CD8^+^ T cells stained for granzyme B (Granzyme B+/CD8a+) after 10-day co-culture (right upper quadrant) with (A) pancreatic cancer organoids, (B) with PMA/ionomycin (positive control) and (C) with healthy pancreatic organoids (negative control).

**Fig. 8:**
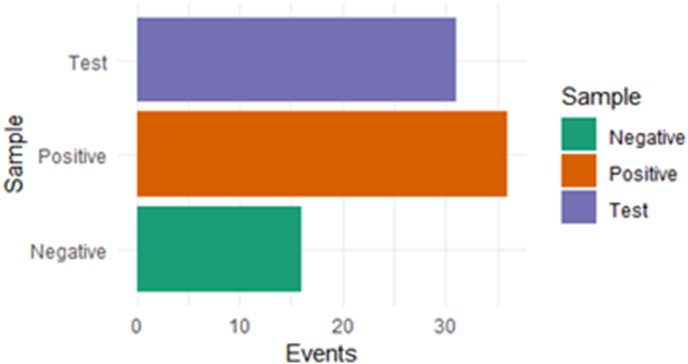
Visual presentation of events observed for PBMCs-turned-CD8^+^ T cells stained for granzyme B after co-culture of PBMCs with pancreatic cancer organoids (test) and healthy organoids (negative control). Positive control: events after PBMC incubation with PMA/ionomycin.

To test whether tumour organoids might be used to obtain tumour-reactive T cells, PBMCs were isolated from an autologous mouse and co-cultured with pancreatic cancer organoids for 10 days. Tumour organoids were pre-stimulated with IFNγ to enhance antigen presentation. Plate-bound anti-CD28 and interleukin-2 (IL-2) were added to provide co-stimulation and to support T cell proliferation, respectively. The tumour reactivity of PBMCs-turned-CD8^+^ T cells was evaluated after 10 days of co-culture with cancer organoids by staining for granzyme B (Fig. 7A). As a negative control, PBMCs were co-cultured for 10 days with healthy pancreatic organoids (Fig. 7C) and stained for granzyme B. As a positive control, PBMCs were incubated with PMA/ionomycin and stained for granzyme B (Fig. 7B). We observed increased staining for granzyme B in PBMCs incubated with tumour organoids compared to those incubated with healthy organoids (Table 1, Fig. 8).

**Table 1:** The number of events observed for PBMCs-turned-CD8^+^ T cells stained for granzyme B after co-culture of PBMCs with (A) pancreatic cancer organoids (test) and (C) healthy organoids (negative control). (B) events after PBMCs were incubated with PMA/ionomycin (positive control).

Specimen	Population	Events
Test (A)	Granzyme B + CD8a+	31
Positive (B)	Granzyme B + CD8a+	36
Negative (C)	Granzyme B + CD8a+	16

### 3.5 Specificity of organoid-reactive T cell responses

**Fig. 9:**
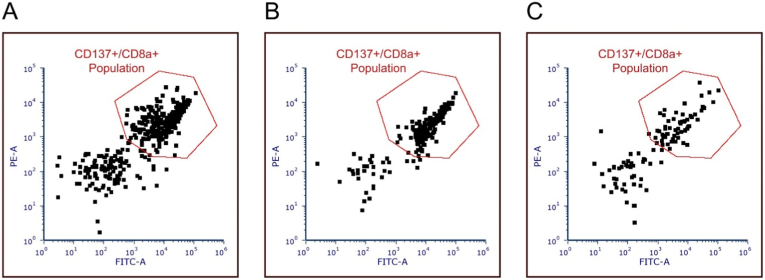
FACS analysis of tumour reactivity of PBMCs-turned-CD8^+^ T cells stained for CD137 (CD137+/CD8a+) after 24-h co-culture (gate in red) with (A) pancreatic cancer organoids, (B) with PMA/ionomycin (positive control) and (C) with healthy pancreatic organoids (negative control). (For interpretation of the references to color in this figure legend, the reader is referred to the Web version of this article.)

**Fig. 10:**
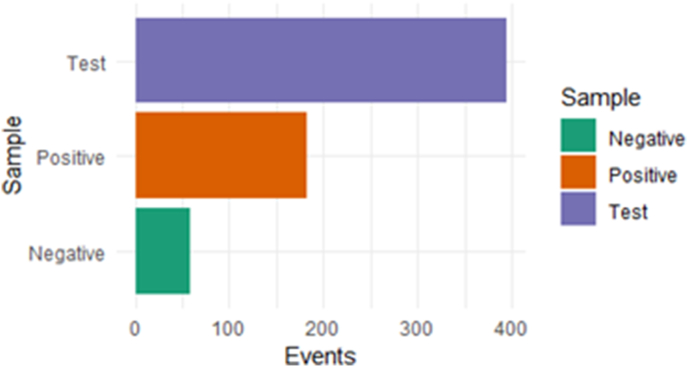
Visual presentation of events observed for PBMCs-turned-CD8^+^ T cells stained for CD137 after co-culture of PBMCs with pancreatic cancer organoids (test) and healthy organoids (negative control). Positive control: events after PBMC incubation with PMA/ionomycin.

It was next evaluated whether the T cell response induced by organoid co-culture was tumour-specific or should be considered an artefact of IFNγ treatment or organoid culture. Towards this goal, PBMC expression of the activation marker CD137 was compared upon stimulation with tumour organoids (Fig. 9A). To more directly assess whether the induced PBMC response was specific for cancer antigens, PBMCs response to stimulation with organoids of healthy pancreatic tissue was tested (Fig. 9C). As a positive control, PBMCs were incubated with PMA/ionomycin mix (Fig. 9B). T cell reactivity induced in 10-day co-culture was significantly restricted to tumour organoids (Table 2). Markedly lower T cell reactivity against healthy tissue was observed (Fig. 10).

**Table 2:** Summary of the number of events observed for tumour reactive PBMCs-turned-CD8^+^ T cells stained for CD137 after co-culture of PBMCs with (A) pancreatic cancer organoids (test) and (C) healthy organoids (negative control). (B) events after PBMCs were incubated with PMA/ionomycin (positive control).

Specimen	Population	Events
Test (A)	CD137 + CD8a+	325
Positive (B)	CD137 + CD8a+	183
Negative (C)	CD137 + CD8a+	59

## 4 Discussion

LSL-Kras^G12D^ Trp53^lox/+^ Pdx1-Cre engineered mouse models were used to investigate pancreatic cancer. These genetically engineered mouse models (GEMMs) developed pancreatic cancer with a six-month latency – cancer growth is expected between four and eight months. Although this mouse model does not represent the most common GEMM for pancreatic cancer, it is known for the development of a well differentiated pancreatic ductal adenocarcinoma (PDAC) with a strong stromal desmoplastic reaction and no metastasis [9]. The use of this specific GEMM is commonly supposed to create a pancreatic cancer tumour microenvironment similar to the human pancreatic cancer tumour microenvironment [10].

After collection of the tumour sample, organoids were generated from both healthy pancreatic tissue and malignant pancreatic tissue. PBMCs were extracted from the blood of both a healthy mouse and a pancreatic cancer GEMM. Pancreatic cancer organoids were then co-cultured with PBMCs in order to establish a potential individualized ex vivo model system to support T cell-based therapies. This is consistent with recent recommendations to explore new models to facilitate the development of patient-specific treatments [7].

T cell-mediated tumour killing is unpredictable due to the heterogeneity of T cell receptors (TCRs) and human leukocyte antigen (HLA), and the unique nature of neo-antigens expressed in human cancers [11]. It is therefore logical to hypothesise that personalized approaches will represent an important advance in immuno-oncology. Most knowledge of T cell-mediated killing of cancer cells has so far come from melanoma studies and less is known in terms of epithelial cancers [12,20].

Dijkstra et al. recently established a transferable approach based on colorectal cancer organoids co-cultured with T cells isolated from patients [4]; using this study as reference, proof of concept is provided here that pancreatic cancer organoids are a useful approach, at least in murine models, for establishing an ex vivo system to support T cell-based therapies and for studying the interactions between cancer cells and T cells. Several groups have recently used co-culture approaches to investigate tumour invasion and interactions between immune cells and cancer organoids. Koikawa et al., for instance, observed that co-culturing PDAC organoids with pancreatic stellate cells – cancer-associated fibroblast (CAF) precursors – led to tumour cell invasion of the surrounding matrix [8]. Holokai et al. found that myeloid-derived suppressor cells interfered with T cell response against both human and murine cancer organoids [6]. Tsai et al. established a 3D culture model including T cells, organoids and CAFs which resulted in increased drug resistance when compared to patient-derived organoids alone [17].

Here, a murine pancreatic cancer organoid culture and a parallel culture of healthy pancreas organoids were established. Co-culture of peripheral blood lymphocytes with pancreatic cancer organoids resulted in significantly increased induction of tumour reactivity in T cells compared to healthy controls, as assessed by intracellular staining of granzyme B. Staining for expression of the activation marker CD137 showed that the T cell responses induced by co-culture were specific to the cancer organoids.

Although the use of cancer co-culture with immune cells is not novel per se, we believe that our approach is methodologically solid, elegant and could be leveraged as a platform for different applications. One possible application is to exploit the co-culture of autologous cancer organoids and T cells as model systems for developing patient-specific immune-oncological treatment regimens and for the investigation of tumour immune networks. Given that tumour organoids include stromal cells, thus re-creating the original tumour microenvironment, they can be considered a sound approach for accurately predicting drug response and resistance as compared to tumour cell lines. Also, establishing tumour organoid cultures from a limited quantity of cancer cells (e.g., needle biopsies), coupled with the potential expansion of tumour-reactive T cells, could enable minimally invasive tumour treatment at multiple time points. For a patient who initially responded and subsequently relapsed, for instance, establishing a co-culture of tumour organoids with autologous T cells from blood might shed light on the underlying cause of relapse. A recent investigation by Zaretsky et al. observed that the paired biopsy strategy is effective in a small cohort of patients with melanoma who relapsed due to the acquisition of JAK1/2 mutations [19]. Another potential application involves the expansion of tumour-reactive T cells from co-culture with tumour organoids for establishing an adoptive T cell therapy or for the identification of tumour-reactive T cell receptors (TCRs) that can be genetically engineered to target cancer cells. This strategy, bypassing the need for surgical specimen resection to isolate tumour infiltrating lymphocytes, could enable a clinically feasible approach for the generation of patient-specific T cells suitable for adoptive T cell transfer. Both applications require further validation and methodological improvement.

## 5 Limitations and future directions

In terms of limitations, firstly we used genetically engineered murine models which, we believe, do not perfectly re-capitulate the pancreatic cancer tumour microenvironment in humans. Organoid model systems need refinement to better mimic cancers, for example by incorporating components of the tumour microenvironment such as fibroblasts and blood vessels. Second, in our study only a single pancreatic cancer GEMM mouse was used. A larger sample size is needed to determine the success rate of this approach. Third, in this study we focus on a pancreatic tumour type with a known and severe mutational load; the possibility of extending this approach to poorly immunogenic tumours requires further testing. Fourth, a more rigorous approach for the validation of pancreatic cancer organoid cells at the molecular level will also need to be considered; for instance, genome sequencing of pancreatic cancer organoids would allow confirmation of the presence of the expected mutations e.g. Kras^G12D^. The molecular specificity of any identified tumour-reactive T cells will remain enigmatic without a deeper understanding of cancer target antigens. Ultimately, further evidence could be provided for the specificity of tumour cell killing activity by autologous T cells by performing a cytotoxicity assay using a green fluorescent apoptosis probe targeting active caspase-3/7.

## 6 Conclusion

In this proof-of-concept study, T cells isolated from murine blood were co-cultured with autologous pancreatic cancer organoids from GEMM, demonstrating the feasibility of obtaining tumour-reactive T cells by co-culturing murine PBMCs with matched murine tumour organoids.

## Funding

This research did not receive any specific grant from funding agencies in the public, commercial, or not-for-profit sectors.

## Declaration of competing interest

The authors declare that they have no known competing financial interests or personal relationships that could have appeared to influence the work reported in this paper.

## References

[1] Cattaneo C.M., Dijkstra K.K., Fanchi L.F., Kelderman S., Kaing S., van Rooij N., van den Brink S., Schumacher T.N., Voest E.E. (2020). Tumor organoid–T-cell coculture systems. Nat. Protoc..

[2] Dahiya D.S., Kichloo A., Singh J., Albosta M., Lekkala M. (2021). Current immunotherapy in gastrointestinal malignancies A Review. J. Invest. Med..

[3] Dangles-Marie V., Pocard M., Richon S., Weiswald L.B., Assayag F., Saulnier P., Judde J.G., Janneau J.L., Auger N., Validire P., Dutrillaux B., Praz F., Bellet D., Poupon M.F. (2007). Establishment of human colon cancer cell lines from fresh tumors versus xenografts: comparison of success rate and cell line features. Cancer Res..

[4] Dijkstra K.K., Cattaneo C.M., Weeber F., Chalabi M., van de Haar J., Fanchi L.F., Slagter M., van der Velden D.L., Kaing S., Kelderman S., van Rooij N., van Leerdam M.E., Depla A., Smit E.F., Hartemink K.J., de Groot R., Wolkers M.C., Sachs N., Snaebjornsson P., Monkhorst K., Haanen J., Clevers H., Schumacher T.N., Voest E.E. (2018). Generation of tumor-reactive T cells by Co-culture of peripheral blood lymphocytes and tumor organoids. Cell.

[5] Fujii M., Matano M., Nanki K., Sato T. (2015). Efficient genetic engineering of human intestinal organoids using electroporation. Nat. Protoc..

[6] Holokai L., Chakrabarti J., Lundy J., Croagh D., Adhikary P., Richards S.S., Woodson C., Steele N., Kuester R., Scott A., Khreiss M., Frankel T., Merchant J., Jenkins B.J., Wang J., Shroff R.T., Ahmad S.A., Zavros Y. (2020). Murine- and human-derived autologous organoid/immune cell Co-cultures as pre-clinical models of pancreatic ductal adenocarcinoma. Cancers.

[7] Horvath P., Aulner N., Bickle M., Davies A.M., Nery E. Del, Ebner D., Montoya M.C., Östling P., Pietiäinen V., Price L.S., Shorte S.L., Turcatti G., Von Schantz C., Carragher N.O. (2016). Screening out irrelevant cell-based models of disease. Nat. Rev. Drug Discov..

[8] Koikawa K., Ohuchida K., Ando Y., Kibe S., Nakayama H., Takesue S., Endo S., Abe T., Okumura T., Iwamoto C., Moriyama T., Nakata K., Miyasaka Y., Ohtsuka T., Nagai E., Mizumoto K., Hashizume M., Nakamura M. (2018). Basement membrane destruction by pancreatic stellate cells leads to local invasion in pancreatic ductal adenocarcinoma. Cancer Lett..

[9] Lee J.W., Komar C.A., Bengsch F., Graham K., Beatty G.L. (2016). Genetically engineered mouse models of pancreatic cancer: the KPC model (LSL-Kras(G12D/+) ;LSL-Trp53(R172H/+) ;Pdx-1-Cre), its variants, and their application in immuno-oncology drug discovery. Curr. Protoc. Pharmacol..

[10] Mazur P.K., Herner A., Neff F., Siveke J.T. (2015). Current methods in mouse models of pancreatic cancer. Methods Mol. Biol..

[11] Pitt J.M., Vétizou M., Daillère R., Roberti M.P., Yamazaki T., Routy B., Lepage P., Boneca I.G., Chamaillard M., Kroemer G., Zitvogel L. (2016). Resistance mechanisms to immune-checkpoint blockade in cancer: tumor-intrinsic and -extrinsic factors. Immunity.

[12] Rosenberg S.A., Restifo N.P. (2015). Adoptive cell transfer as personalized immunotherapy for human cancer. Science (80-.).

[13] Sahin U. (2018). Studying tumor-ReacTive T cells: a personalized organoid model. Cell Stem Cell.

[14] Sharma P., Hu-Lieskovan S., Wargo J.A., Ribas A. (2017). Primary, adaptive, and acquired resistance to cancer immunotherapy. Cell.

[15] Siegel R.L., Miller K.D., Jemal A. (2020). Cancer statistics, 2020. CA. Cancer J. Clin..

[16] Stevanović S., Draper L.M., Langhan M.M., Campbell T.E., Kwong M.L., Wunderlich J.R., Dudley M.E., Yang J.C., Sherry R.M., Kammula U.S., Restifo N.P., Rosenberg S.A., Hinrichs C.S. (2015). Complete regression of metastatic cervical cancer after treatment with human papillomavirus–targeted tumor-infiltrating T cells. J. Clin. Oncol..

[17] Tsai S., McOlash L., Palen K., Johnson B., Duris C., Yang Q., Dwinell M.B., Hunt B., Evans D.B., Gershan J., James M.A. (2018). Development of primary human pancreatic cancer organoids, matched stromal and immune cells and 3D tumor microenvironment models. BMC Cancer.

[18] Versteijne E., Suker M., Groothuis K., Akkermans-Vogelaar J.M., Besselink M.G., Bonsing B.A., Buijsen J., Busch O.R., Creemers G.J.M., van Dam R.M., Eskens F.A.L.M., Festen S., de Groot J.W.B., Koerkamp B.G., de Hingh I.H., Homs M.Y.V., van Hooft J.E., Kerver E.D., Luelmo S.A.C., Neelis K.J., Nuyttens J., Paardekooper G.M.R.M., Patijn G.A., van der Sangen M.J.C., de Vos-Geelen J., Wilmink J.W., Zwinderman A.H., Punt C.J., van Eijck C.H., van Tienhoven G. (2020). Preoperative chemoradiotherapy versus immediate surgery for resectable and borderline resectable pancreatic cancer: results of the Dutch randomized phase III PREOPANC trial. J. Clin. Oncol..

[19] Zaretsky J.M., Garcia-Diaz A., Shin D.S., Escuin-Ordinas H., Hugo W., Hu-Lieskovan S., Torrejon D.Y., Abril-Rodriguez G., Sandoval S., Barthly L., Saco J., Homet Moreno B., Mezzadra R., Chmielowski B., Ruchalski K., Shintaku I.P., Sanchez P.J., Puig-Saus C., Cherry G., Seja E., Kong X., Pang J., Berent-Maoz B., Comin-Anduix B., Graeber T.G., Tumeh P.C., Schumacher T.N.M., Lo R.S., Ribas A. (2016). Mutations associated with acquired resistance to PD-1 blockade in melanoma. N. Engl. J. Med..

[20] Zheng C., Sun Y.H., Ye X.L., Chen H.Q., Ji H. Bin (2011). Establishment and characterization of primary lung cancer cell lines from Chinese population. Acta Pharmacol. Sin..

