# Supplementary material for: Generation of murine tumour-reactive T cells by co-culturing murine pancreatic cancer organoids and peripheral blood lymphocytes
Source: Biochem Biophys Rep. 2022 Oct 9;32:101365. doi: 10.1016/j.bbrep.2022.101365 (PMC9552097; doi:10.1016/j.bbrep.2022.101365)
Supplement: Multimedia component 1 [file mmc1.docx]

**Appendices**

**Key resources table**

| **REAGENT** | **SOURCE** | **IDENTIFIER** |
| --- | --- | --- |
| **Antibodies** | | |
| Anti-mouse CD28 | BioLegend | Cat No. 102101 |
| Anti-mouse CD8a (FITC) | BioLegend | Cat No. 100705 |
| Anti-mouse CD137 (PE) | BioLegend | Cat No. 106105 |
| Anti-mouse TCRb (APC) | BioLegend | Cat No. 109211 |
| Anti-mouse I-A/I-E (PerCP) | BioLegend | Cat No. 107625 |
| Anti-human/mouse Granzyme B (PE) | BioLegend | Cat No. 372207 |
| **Biological Samples** | | |
| Murine tumour and normal pancreatic tissue | Yamaguchi University |  |
| Murine blood | Yamaguchi University |  |
| **Chemicals, peptides and recombinant proteins** | | |
| B27 Supplement | Gibco | Cat No. 17504-044 |
| N-Acetylcysteine | Sigma-Aldrich | Cat No. A9165-5G |
| Nicotinamide | Sigma-Aldrich | Cat No. N0636 |
| Murine Recombinant EGF | Peprotech | Cat No. AF-315-09 |
| A83-01 | Tocris | Cat No. 2939 |
| Y-27632 | Sigma-Aldrich | Cat No. Y-0503 |
| Murine Recombinant FGF-2 | Peprotech | Cat No. [AF-450-33](javascript:TrackClick('/gb/recombinant-murine-fgf-basic-2',%20%7b%20list:%20%20'Search%20Results',name:%20'Animal-Free%20Recombinant%20Murine%20FGF-basic',id:%20'AF-450-33',position:%203%7d);) |
| Matrigel (Growht Factor Reduced) | BD | Cat No. 356230 |
| Collagenase type II | Sigma-Aldrich | Cat No. C6885 |
| Lymphoprep | Stemcell | Cat No. 07801 |
| Advanced DMEM-F12 (aDMEM/F-12) | Gibco | Cat No. 12634-028 |
| Human serum AB | Sigma-Aldrich | Cat No. H5667-20ML |
| Penicillin/streptomycin | Gibco | Cat No. 15070063 |
| Ultraglutamine type I | Lonza | Cat No.BW17-605E |
| HEPES | Gibco | Cat No. 15630-056 |
| TrypLE Express | Gibco | Cat No.12604-013 |
| Human serum AB | Sigma-Aldrich | Cat No. H6914 |
| RPMI 1640 | Gibco | Cat No. 11875093 |
| Human recombinant interferon gamma (INFγ) | Peprotech | Cat No. 300-02 |
| GolgiPLUG (Brefeldin A) | BD | Cat No. 555029 |
| Phorbol 12-myristate 13-acetate (PMA) | Sigma-Aldrich | Cat No. 19-144 |
| Ionomycin | Sigma-Aldrich | Cat No. I9657 |
| Primocin | Invivogen | Cat No. PML-40-60 |
| Dispase type II | Sigma-Aldrich | Cat No. D4693 |
| Interleukin-2 | Novartis | Proleukin^®^ |
| **Critical Commercial Assays** | | |
| Cytofix/Cytoperm kit | BD | Cat No. 554722 |
| Perm/Wash | BD | Cat No. 554723 |
| **Experimental Models: Cell lines** | | |
| L-Wnt3a cell line | Protocol 1 |  |
| R-spondin producer cell line | Protocol 2 |  |
| Noggin producer cell line | Protocol 3 |  |
| **Software and algorithms** | | |
| FlowJo version 1.0 |  | https://www.flowjo.com/ |
| FCSvExpress version 7 |  | https://denovosoftware.com |
| ImageJ version 1.50i |  | https://imagej.nih.gov/ij/index.html |

**Protocol 1**

Preparation of L-Wnt3a-conditioned medium (timing 14 days approximately):

1. Culture L-Wnt3A cells in a 100-mm dish until the cells become confluent

2. Trypsinize the cells with Trypsin-EDTA, and passage the cells into three 175-mm2 flasks. Add 25 ml of 10% (vol/vol) FBS/DMEM supplemented with 125  ml−1 zeocin

3. Incubate the cells at 37 °C for 3–4 d until they become confluent. Trypsinize and passage the cells into 15 175-mm2 flasks. Add 25 ml of 10% (vol/vol) FBS/DMEM.
crItIcal step Do not add zeocin to the culture medium for this and the following steps.

4. Incubate the cells at 37 °C for 2–3 d until they become confluent. Trypsinize and passage the cells into 30 175-mm2 flasks. Culture the cells with 25 ml of 10% (vol/vol) FBS/DMEM.

5. Collect the conditioned medium after 1 week of incubation, and centrifuge it at 300g for 5 min. Sterilize the conditioned medium with a vacuum filter.

6. Transfer the conditioned medium to 50-ml centrifuge tubes, and store the tubes at −20 °C until use.

The conditioned medium can be stored at −20 °C for 6 months without loss of activity. The thawed conditioned medium can be kept at 4 °C for up to a month. Do not freeze it again after thawing.

**Protocol 2**

Preparation of R-spondin-conditioned medium (timing: 14 days approximately)**:**

A. Thawing the cells (CultrexR HA-R-spondin 1 Fc 293T Cells; Cat# 3710-001-01 Lot # 34147K5)

1. Thaw the cells as other cell lines are thawed.

2. Start culture using a Basal Growth Medium [DME 500 mL; FBS 55 mL; Penicillin & Streptomycin (x100) 5.5 mL; GultaMAX(x100) 5.5 mL in sterile T25 (25 cm^2^) Tissue Culture Flask.

3. Change medium the next day.

B. Passaging the cells.

Note: HA-R-spondin 1 Fc 293T Cells may be sequentially passaged under selection into larger culture vessels to expand cell numbers. Cultures should be maintained at density between 40-90 % confluent for optimal growth and survival.

C. R-spondin1–conditioned medium

1. Culture the R-spondin1–producing cell line in a 100-mm dish with 10% (vol/vol) FBS/DMEM until the cells become confluent.

2.  Trypsinize the cells with Trypsin-EDTA, and passage the cells into three 175-mm2 flasks. Add 25 ml of 10% (vol/vol) FBS/DMEM supplemented with 300 μg /ml zeocin.

3.　Incubate the cells at 37 °C for 3–4 d until they become confluent. Trypsinize and passage the cells into 15 175-mm2 flasks. Add 25 ml of 10% (vol/vol) FBS/DMEM.

4.　Incubate the cells at 37 °C for 2–3 d until the cells become confluent. Trypsinize the cells and collect the cells with 10 ml of basal medium. Centrifuge the cells at 300*g* for 5 min at 4 °C and discard the supernatant. Passage the cells into 15 175-mm2 flasks, and culture them with 50 ml of basal medium.

5. Collect the conditioned medium after 1 week of incubation, and centrifuge it at 300*g* for 5 min at 4 °C. Sterilize the conditioned medium with a vacuum filter.

6. Make aliquots of 2 ml in 2-ml cryovials and store them at −20 °C until use.

The conditioned medium can be stored for 6 months without loss of activity. Use the medium immediately after thawing, and do not freeze it again.

**Protocol 3**

Preparation of Noggin-conditioned medium (timing 10 days approximately):

1. Preheat HEK293 growing medium to 37 °C.
2. Thaw a vial of HEK293-mNoggin-Fc cells in a 37 °C water bath until a small clump of ice remains.
3. Transfer the cells to a 15-mL Falcon tube and add up to 10 mL of preheated HEK293 growing medium.
4. Centrifuge (300g, for 5 min at RT) and discard the supernatant.
5. Resuspend the cell pellet in 50 mL of selection medium and transfer to a T-175 culture flask.
6. Culture cells until confluency with the T-175 flask in a flat position.
7. Aspirate the medium and wash flask with 20 mL of PBS.
8. Collect cells by trypsinizing for up to 5 min with 5 mL of TrypLE Express at 37 °C.
9. Transfer cells to a 50-mL Falcon tube and add up 50 mL of PBS.
10. Centrifuge (300g, for 5 min at RT) and discard the supernatant.
11. Resuspend cells in HEK293 growing medium and split into 6× T-175 flasks. Culture five flasks in 50 mL of HEK293 growing medium and one flask in 50 mL of selection medium.
12. When cells reach confluency, replace medium with 50 mL of Ad-DF+++.
13. Collect the conditioned medium after 1 week and centrifuge (300g, for 5 min at RT) to pellet cells.
14. Collect the supernatant and filter through a 0.2-μm filter unit.
15. Store the supernatant in aliquots of 50 mL at −20 °C. Conditioned medium can be stored for up to 6 months at −20 °C and up to 2 weeks at 4 °C.
16. Use the cells cultured in the single flask with selection medium to repeat the procedure, or freeze the cells in small aliquots for later use.
